# Correlation Study of Honey Regarding their Physicochemical Properties and Sugars and Cyclitols Content

**DOI:** 10.3390/molecules25010034

**Published:** 2019-12-20

**Authors:** Ileana Andreea Ratiu, Hossam Al-Suod, Małgorzata Bukowska, Magdalena Ligor, Bogusław Buszewski

**Affiliations:** 1 Interdisciplinary Centre of Modern Technologies, Nicolaus Copernicus University, Wileńska 4, 87-100 Toruń, Poland; andreea_ratiu84@yahoo.com (I.A.R.);; 2 Department of Environmental Chemistry and Bioanalytics, Faculty of Chemistry, Nicolaus Copernicus University, Gagarina 7, 87-100 Toruń, Poland; 3 Babeş-Bolyai University, Faculty of Chemistry and Chemical Engineering, 11 Arany Janos, RO-400028 Cluj-Napoca, Romania

**Keywords:** honey, sugars and cyclitols, physicochemical properties, correlation analysis

## Abstract

Honey is a natural sweetener, with an osmotic effect on microorganisms due to the increased sugar content and low amount of water. Cyclitols are minor constituents of honey. They play a defensive role in plants against unfavorable environmental conditions. Honey’s physicochemical properties can vary, resulting in a wide range of colors, flavors, scents, antioxidant activity, dissimilar values of pH, acidity, electrical conductivity, etc. Some literature regarding correlation between honey types is already available, but a comprehensive study displaying an ample evaluation of multifarious aspects is still needed. This study focuses on the correlation between 18 honey types, originating from 10 countries, collected during four years, summarizing a total of 38 samples. A total of 6 physicochemical properties and 18 target components (sugars and cyclitols) were considered as variables. A correlation analysis is presented between the investigated parameters and between honey types, together with the statistical analysis which allowed for observation of the clusters’ distribution according with the investigated variables.

## 1. Introduction

Honeybees (*Apis mellifera* L.) are herbivorous pollinator insects, consuming nectar and pollen throughout their life cycles. The nectar is primarily an energy source which, in addition to sugars contains various other components with important nutritional properties. However, honey is a natural product, known for its antibacterial properties [1] and medical uses [2], with osmotic effect due to the increased sugar content and low amount of water [3]. Honey is quite acidic (with values between 3.2 and 4.5), a fact that inhibits the pathogens, while the hydrogen peroxide produced due to glucose oxidase also helps the preservation of this foodstuff [4]. Besides being a high-energy carbohydrate product with easy digestible sugars, such as those in fruits, honey contains around 180 substances, including: amino acids, enzymes, proteins, vitamins, minerals, phenolic compounds, etc [5]. The properties and composition of honey are dependent on its geographical origin, type of flowers, harvesting season, environmental factors, and treatments administered by beekeepers [6]. 

Cyclitols (sugar alcohols) are additional minor constituents of honey, and are widely unexplored compared with those mentioned previously. Cyclitols are important antioxidant, anti-cancer and anti-inflammatory agents [7]. However, they are secondary metabolites, naturally occurring in plant material, playing an important role in plant self-defense against unfavorable environmental conditions [8]. Moreover, cyclitols are responsible for the cell’s good functioning, cell wall formation, phosphate storage, and osmoregulation. In the pharmaceutical industry, they are used in the treatment of psychiatric dysfunctions, panic attacks, depression, or obsessive-compulsive disorders [8]. It is seldom acknowledged that, despite its bactericidal effect [3], honey can sometimes contain pathogens (bacteria, fungi, or yeasts) that can withstand concentrated sugar and acidity [9]. The pathogens can arrive in honey either via pollen, or due to improper honey manipulation and storage. Moreover, pesticides or their residues can often be present in honey [10].

Some of the chromatographic techniques generally used for the composition analysis of honey samples, can also be employed for the analysis of sugars and cyclitols, the most widely used being high performance liquid chromatography (HPLC) [11,12]. Gas chromatography with mass spectrometry (GC-MS) is quite often used as well, but this technique involves a derivatization step [13,14,15]. In terms of the sample preparation for the detection of cyclitols and sugars, honey samples can be easily handled, since just a dilution in water is required. Conversely, for the analysis of cyclitols and sugars from plant material, an elaborated procedure is required in terms of both, the solvent used and the extraction techniques [15,16,17,18,19,20].

**Figure 1:**
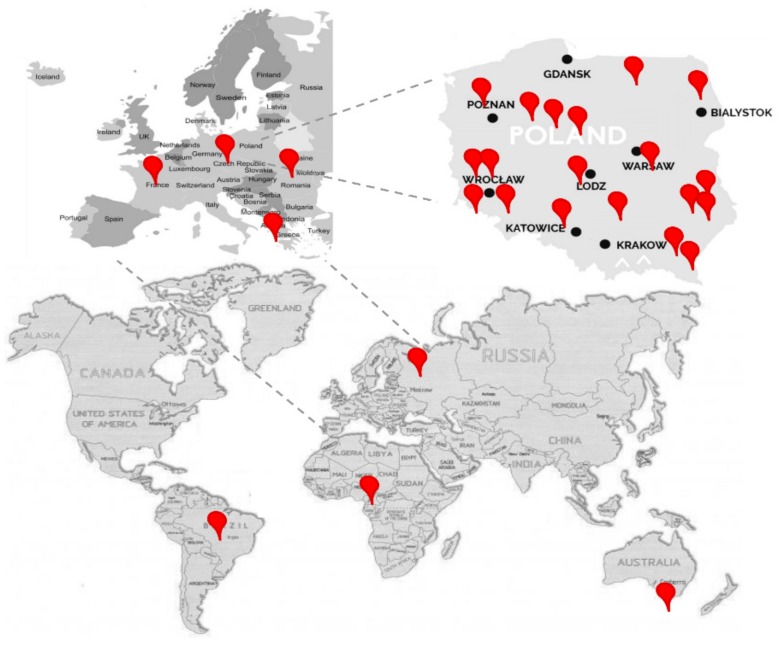
Map presenting the origin of samples.

The current study presents a comprehensive comparison of 18 types of honey, coming from 10 countries and collected over four years in terms of the correlation between their assortment as well as the content of sugars and cyclitols. A map presenting the samples origins is presented in Figure 1. As variables, six physico-chemical properties (Pfund value, color, pH, acidity, electric conductivity, and antioxidant activity), along with seven sugars and 11 cyclitols detected and quantified in honey samples were used. Consequently, a complex correlation analysis was developed between both the investigated parameters and honey types. Our obtained results concluded that there is a relevant correlation between different physical properties and types of honey as well. The samples cultivated in 2015 and 2016 a presented distinctive correlation compared with those harvested in 2017 and 2018, due to the natural changes which occur in honey during the storage period. Clusters analyses based on detected sugars and cyclitols segregated the samples based on the strength of the total amount of detected targets. Hierarchical clustering analyses with respect to the 23 variables selected, led to the formation of 11 groups, including five main clusters with high significance and another six more, with lower levels of significance, or simply fused together in an aleatory way.

## 2. Results and Discussion 

### 2.1. Role and Assessment of Physico-Chemical Properties of Honey

**Figure 2:**
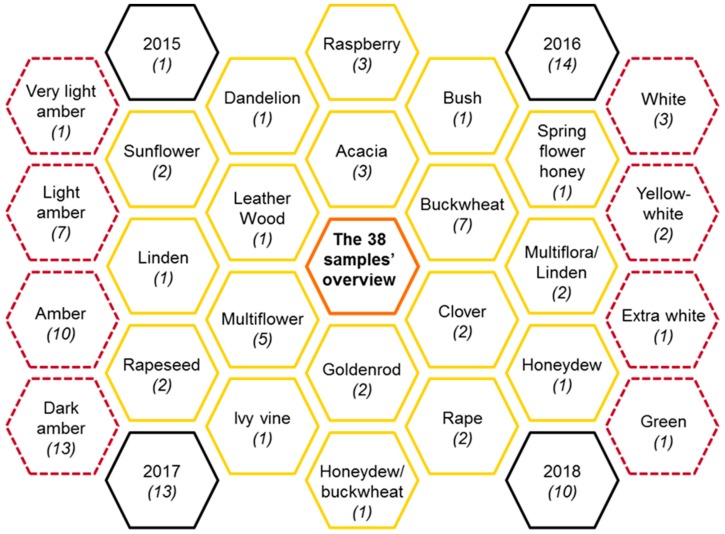
The distribution of the 38 samples, in terms of honey type, color and cultivability year.

The investigated samples were collected from 10 different countries, over four years (2015 to 2018), summarizing 18 varieties. Generally, the honey color was amber, ranging from a very light to dark amber shade. However, less common color like: white, extra white, yellow-white or green were the subject of analyses. Pfund value (mm) was between 10.2 in the case of extra white Acacia honey and 514.55 for the green honey of Ivy vine (corresponding to dark amber, according with Pfund scale). In Figure 2 a distribution of the 38 samples in terms of honey type, colors and cultivability year was drawn. The number for a specific category is presented between brackets, each set summarizing the number 38. 

The electrical conductivity of honey is connected with the content of mineral and organic acids. It is part of the routine analysis for honey control and often a method involved for honey origin identification [21]. However, the maximum approved value of electrical conductivity for edible honey is 800 S/cm, (the equivalent of 0.8 mS/cm), as stipulated by the EU Directive [22]. Four samples presented in Table 1, namely samples 2, 22, 32, and 38, exceeded the maximum allowed value of 0.8 mS/cm. However, electrical conductivity ranged from 0.041± 0.02 (in spring flowers honey, collected from Poland, 2018) up to 1.221 ± 0.05 (Linden/Multiflora honey, from Poland, 2016).

Honey acidity is linked with the presence of organic acids naturally occurring in this foodstuff, which is closely connected to, and balanced with the content of lactones, esters, phosphates ions, sulfates ions and chlorides ions [21]. According with the EU Directive [22], the maximum allowed value of acidity is 50 meq/kg. An increased value of acidity denotes the beginning of the fermentation process, through which the produced alcohols are transformed into organic acids [21]. In eight of the 38 investigated honeys (samples 5, 14, 25, 26, 28, 29, 34, and 35) the maximum acidity value allowed by the European standards was exceeded (as presented in Table 1). These samples belong to the categories: multiflora, buckwheat, ivy vine, and honeydew/buckwheat. Moreover, all of them had a dark amber color. Due to the observation that four out of seven buckwheat honeys, two out of five multiflora honeys, and only one sample from both honeydew/buckwheat and ivy vine honey presented increased values of acidity, we concluded that these honeys are more easily subject to fermentation, and/or that it is generally possible that in dark honeys the fermentation process is facilitated faster. This hypothesis was confirmed by other researchers as well [21]. On the opposite side were the white, extra-white, yellow-white and light amber honeys (rape, linden, acacia, clover, and sunflower) which presented considerably low values of acidity, generally ranging from 12 to 24 meq/kg. These findings indicate that in the honeys with light colors considerably lower contents of organic acids are present in comparison with dark honeys. Nevertheless, it was highlighted that the content of organic and amino acids, on which the acidity is dependent, are contingent on the botanical origin of honey [23,24]. In our investigated samples the acidity ranged from 12 to 114 meq/kg. Notably, the two extreme values were recorded for the same type of honey, a multiflora variety coming from Greece and Kameron respectively, denoting the clear degradation of the sample from Kameron.

The honey pH value is connected with the existence and growth of microorganisms. The EU Directive [22] does not impose a maximum allowed value for honey pH, however, a low pH will prevent microbiological spoilage. In our samples, the pH was ranging from 3.20 ± 0.01 to 4.49 ± 0.01, a fact that apparently denotes the absence of bacteria. 

Regarding antioxidant activity, there are a couple of methods used for testing this parameter in honey, such as: free radical scavenging activity (DPPH), ferric reducing/antioxidant power (FRAP), oxygen radical absorbance capacity (ORAC), ascorbic acid content (AEAC), and Trolox equivalent antioxidant activity (TEAC). Notwithstanding, each one of them allows the measurement of a different group of antioxidants, and consequently it cannot be affirmed that there is an ideal one which can fully evaluate the antioxidant activity [25]. It is supposed constituents in honey like: flavonoids, phenolic acids, vitamins, enzymes, as well as a small amount of mineral content, particularly copper and iron can be responsible for antioxidant activities [26]. TEAC antioxidant activity (expressed in trolox equivalent antioxidant activity) has been tested in this study. Values between 2.53 (Acacia honey coming from Romania) and 7.03 (a mixture of honeydew and buckwheat, coming from a private beekeeper from Janowiec, Lubelskie, Poland) were obtained. It was observed that the dark amber honey generally showed higher antioxidant activity compared with the honey with lighter color, as presented in Table 1.

### 2.2. The Content of Sugars and Cyclitols in the Investigated Honey Samples

**Figure 3:**
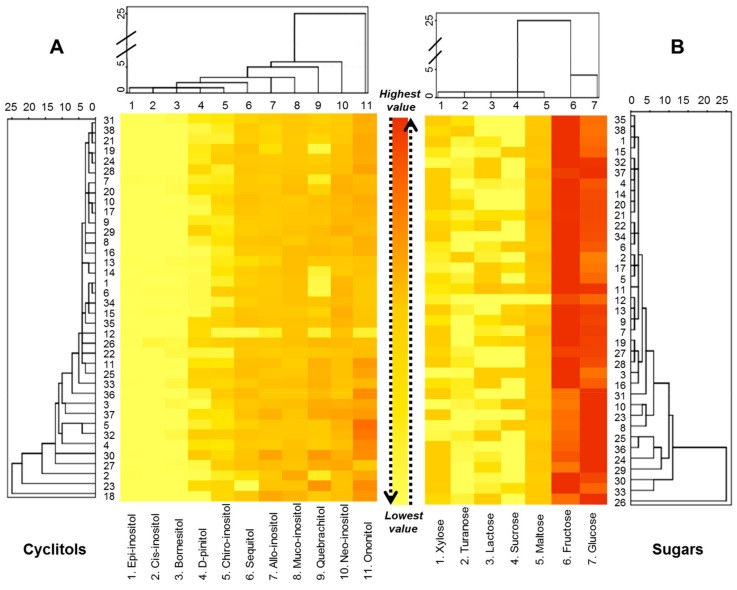
Heat maps presenting the quantity of cyclitols (part **A**) and sugars (part **B**) detected in honey samples. The sample numbers from the vertical dendrograms were allotted similar to those presented in Table 1.

The targets detected (sugars and cyclitols) were simultaneously separated in one chromatographic run by a single GC column. Some components (fructose and glucose) appeared as different isomers in the form of two or three peaks. The peaks of fructose can be identified with α-furanose, β-furanose, and β-pyranose, while the D-glucose peaks are represented by α-pyranose and β-pyranose, as confirmed by other researchers previously [27]. In Figure 3, a heat map combined with a dendrogram is presented for cyclitols (part A) and sugars (part B). The heat map was built to express a snapshot of the quantified concentration of sugars and cyclytols in honey samples. The real amounts are expressed in mg/g and highlighted in Supplementary Table S1. Just the concentrations of cyclitols and main sugars are shown (glucose, fructose, and maltose) in Supplementary Table S1. The hierarchical clustering model based on detected cyclitols (Figure 3A, horizontal part) highlighted the formation of six main clusters. Generally, *epi*-inositol, *cis*-inositol, bornesitol, D-pinitol and *chiro*-inositol were detected in lower amounts compared with other cyclitols, or not detected at all in some samples. Moreover, they fused together in one cluster with similar distance levels, as shown in the left part of Figure 3A. On the other extreme were grouped: ononitol, neo-inositol, quebrachitol and *muco*-inositol, which presented a lower level of similarities compared with those previously mentioned. Nevertheless, they were present in all investigated samples. The lowest total amount of cyclitols (8.38 mg/g) was detected in sample no 12, Sunflower honey, collected in 2016 in Poland and the highest total amount (59.51 mg/g) in sample no 30, Spring flower, from Poland, 2018. However, we could not conclude that the detected amount of cyclitols is connected with honey type while, for example, in the case of Buckwheat honey, for which seven samples were analyzed, the detected quantities ranged between 14.49 (sample no 14) up to 50.58 (sample no 37). Other examples, such as the case of multiflora, raspberry or acacia honey, can be added to this one.

In the case of sugar concentrations (part B), it is worth mentioning that fructose and glucose were the most important sugars quantified, present in all samples in far higher concentration compared with others. As can be observed in the horizontal dendrogram of sugars, glucose and fructose clustered separately from other sugars. Fructose was detected in greatest proportion than glucose, except in some honeys such as rape, dandelion, ivy vine, goldenrod, three form seven buckwheat honeys and two from five multiflora honeys. Notwithstanding, some other researchers reported as well that the glucose is higher than the fraction in rape and dandelion honey, fact that causes the rapid crystallization of honey [28]. Regarding the quantities detected, fructose amount was between 243.3 ± 1.99 mg/g (in sample 8) and 422.65 ± 1.64 mg/g (in sample 3). Glucose amount was between 218.3 ± 0.77 mg/g (in sample 34) up to 449.9 ± 1.35 mg/g (in sample 29).

The dendrograms presented in the vertical parts showed the formation of many clusters, but no clustering according with the honey types was observed. However, the samples alignment corresponded broadly to the total quantity detected. Consequently, alignment the samples, from up to down presented an increasing trend according with the total amount of detected sugars or cyclitols respectively. Each number presented in the vertical dendrograms corresponds to one honey sample and was allotted similar with those presented in Table 1.

### 2.3. Correlation Analyses

In order to run the correlation analysis for the investigated samples, two different approaches were used: non-parametric tests (Spearman correlation) and parametric tests (Pearson correlation). The decision regarding the method chosen was made based on the available data set. However, the obtained correlation for both approaches is presented below.

#### 2.3.1. Correlation Between the Investigated Variables

**Figure 4:**
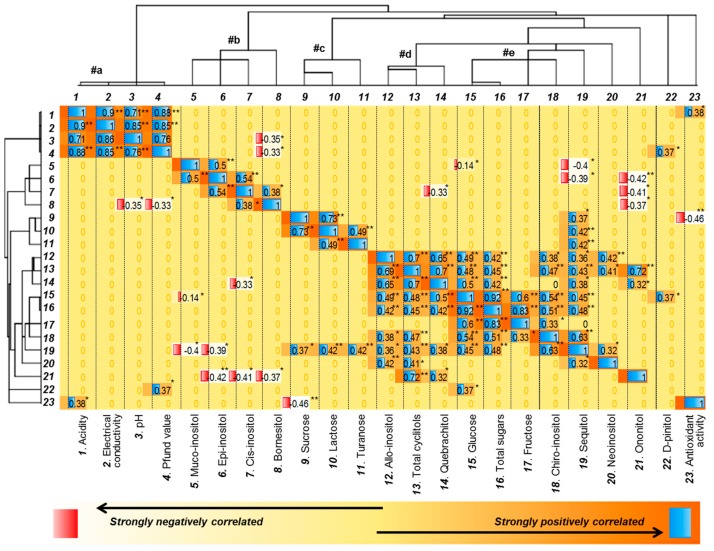
Heat map presenting correlation between the investigated variables together with hierarchical clusters analyses, where ** = correlation significant at the 0.01 level, * = correlation significant at the 0.05 level, a, b, c, d, e = label of the main clusters.

In the case of the correlation between investigated variables, the determined physico-chemical values, total amount of sugars, total amount of cyclitols and the individual targets quantified (seven sugars and 11 cyclitols) of each sample were used. For this data set we chose a non-parametric Spearman correlation, which presents a monotonic relationship between dissimilar variables and is suitable to compare samples with various measurement units. Maltose was the only one variable which did not present correlation with others, and consequently it was removed from the matrix. The correlation between the investigated variables is presented in Figure 4 in the form of a heat map combined with a dendrogram of clusters’ analysis, which shows the formation of five main clusters (which were labeled from #a to #e).

By examination of the relationships between the mentioned groups, a strongly positively correlation related to physical properties: acidity, electrical conductivity, pH and Pfund value, r(21) = 0.76 up to 0.90, *p* = 0.01 was highlighted. Moreover, hierarchical cluster analyses presented all four physical properties fused together in one cluster with similar distance level (cluster #a, Figure 4). Positioned at the opposite site in a rather arbitrarily manner was the cluster corresponding to antioxidant activity. Low correlation was found between acidity and antioxidant activity r(21) = 0.38, *p* = 0.05. Furthermore the antioxidant activity was moderately negative correlated with the sucrose level. This finding indicates that a high level of sucrose will decrease the antioxidant activity potential, while a high acidity will increase the antioxidant activity. Another main cluster (#b) with almost the same level of similarity was segregated from *muco*-inositol, *epi*-inositol, *cis*-inositol and bornesitol. *Muco*-inositol was moderately positive correlated with *epi*-inositol, which in its turn was moderately positive correlated with *cis*-inositol. *Cis*-inositol was howsoever positively related to bornesitol. Moreover, the four mentioned targets have been observed to have low negative correlation with some other compounds (Figure 4).

Two more equal groups of clusters were formed by: sucrose, lactose, turanose (#c) and *allo*-inositol, total cyclitols, and quebrachitol (#d). They presented various low to strong correlations both positively and negatively, between them and with other targets, as presented in Figure 4. Finally, the last main cluster (#e) was formed by segregation of glucose with total sugars (which presented the same level of similarity) and fructose with *chiro*-inositol. A generally low (r(21) = 0.32, *p* = 0.05) up to very strong correlation (r(21) = 0.92, *p* = 0.01) was found between the targets which formed the clusters #c, #d and #e. Figure 4 shows too the formation of other secondary clusters with higher distance levels, which presented lower similarities with the five discussed previously, or they simply fused together in an arbitrary way. As a general conclusion, these findings indicate that there is relevant correlation between honey’s properties, even if these characteristics are far different and in appearance not linked-up with each other.

#### 2.3.2. Correlation Between Honey Types

**Figure 5:**
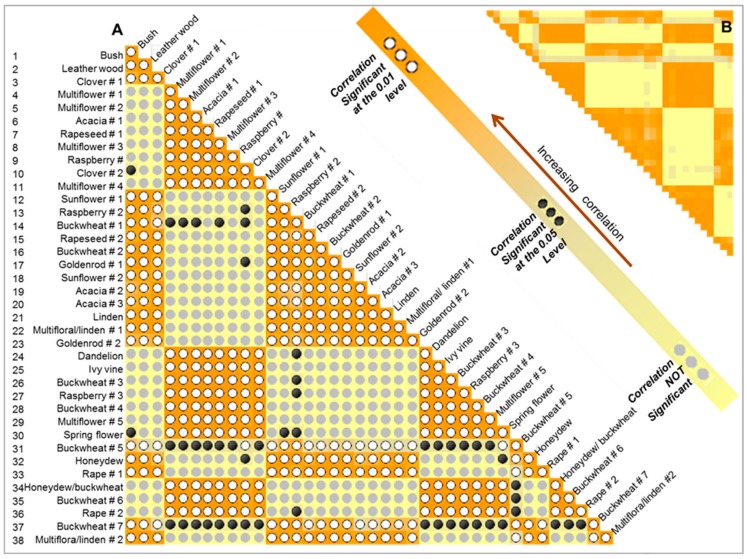
Correlation matrix presenting the significance between honey types (part **A**) and heat map highlighting the difference in correlation values (part **B**).The numbers from 1 to 38 are similar with those presented in Table 1.

Pearson moment product correlation was used to highlight the similarities between honey types, while as variables we considered the 38 investigated samples. The Pearson test was chosen because it is a parametric statistical tool expecting a linear correlation between the investigated variables, suitable when those variables are coming from the same source, and/or they have the same measure unit. The correlation analysis is presented in Figure 5.

In Part A, the correlation matrix is shown presenting the level of significance, while a mirror of this matrix in a heat map form is designed in Part B, in order to highlight the difference between the correlation values. However, we can confirm that the correlation values significant at 0.01 level (light dots) were between r(36) = 0.7 to 0.99, which pointed to a strong to very strong correlation. Moreover, the correlation values significant at a 0.05 level (dark dots), ranged from r(36) = 0.41 to 0.65, denotative of a moderate correlation.

By analyzing the matrix correlation, it was observed that samples 1 to 3, originating from Australia, were generally not correlated with samples 4 to 11, originating from different European countries, Brazil, Kameron or Russia. However, a correlation between Australian samples and some samples coming from Poland did exist, as presented in Figure 5. It was observed that nine out of twelve samples (corresponding to the samples no 12 to 23), which presented strong correlation with the samples from Australia were cultivated in 2015 and 2016.

Notably that all the samples coming from Lenah Valley, Tasmania, Australia were cultivated in 2016, while the other non-correlated samples from European countries, Brazil, Kameron, or Russia were all cultivated in 2017 and 2018. Going forward, we observed that the non-correlated samples (24–30 and 34–36) were all cultivated in 2017 and 2018. Generally, we observed that the samples cultivated in 2015 and 2016 were not correlated with those harvested in 2017 and 2018. However, some notable exceptions were remarked. Samples 14, 31, and 37, belonging to buckwheat type, cultivated in 2016 presented moderate up to very strong correlation with almost all investigated samples. Moreover, clover and spring flower types presented some correlation with all categories as well. All these findings may indicate that the samples cultivated in the same year present a strong correlation, or that during storage honey can change its composition and properties.

No studies were found to confirm or disprove the correlation related to year of cultivability. Instead, other researchers confirmed that during prolonged storage time the honey changes its composition and characteristics. Thus, the literature study outlined the changes in sugar composition, accounting for increasing, and decreasing amounts, as well as sugar degradation and conversion in furans derivatives [29]. Some of the changes occuring in honey during storage that may influence nutritional and sensory properties can be associated with the Maillard reaction, which occurs either slowly during storage, or rapidly by heating. The Maillard reaction is a chemical response between a reducing sugar and a primary amino group, resulting in browning and reduction of nutritional value inhoney [5]. Strecker degradation, another reaction occuring in honey, contributes to the loss of amino acids [5]. Alcohol concentration can increase during storage as well. Their occurrence is generally due to lipid oxidative degradation or reduction processes catalyzed by aldehyde reductase from honey contaminated with yeasts, molds or bacteria [30,31]. Another pathway of alcohols occurence in honey is the transformation of hydrocarbons into smaller molecules due to oxidative processes [30]. The oxidation of fatty acids in honey, especially linoleic and linolenic acids, results in the formation of aldehydes and ketones creating a rancid flavor [31]. However, other constituents reported to undergo changes during storage are: proteins, organic acids, vitamins, minerals, phenolic compounds and volatile compounds [5]. One study regarding Lithuanian honey stability, in terms of the chemical classes described above, concluded that qualitative and quantitative changes started to be evident only after three months of storage. Physical properties such as consistency, crystallization, and rheology were also revealed to be subject to changes during storage in Lithuanian honey [31].

## 3. Materials and Methods

### 3.1. Honey Samples Collection and Chemicals Involved

Most of the honey samples were collected from different regions of Poland (27 samples) from private beekeepers and manufacturers. Other samples were collected from Australia (3 samples), Romania (2 samples), Greece, Kameron, France, Czech Republic, Russia and Brazil (1 sample from each mentioned country). A full list of samples, including place of cultivation, year, manufacturer, and their physicochemical properties is presented in Table 1. 

Standard purity ≥95% D-pinitol, *myo*-inositol, D-*chiro*-inositol, ononitol, bornesitol, *allo*-inositol, *cis*-inositol, *epi*-inositol, D-glucose, D-fructose, D-maltose, lactose, D-sorbitol, and D-(+)-turanose and trimethylsilylimidazole (TMSI) were purchased from Sigma-Aldrich (St. Louis, MO, USA). Standards of sucrose, xylose, quebrachitol, *neo*-inositol, *muco*-inositol with purity ≥98%, and pyridine were bought from Avantor (Gliwice, Poland). DPPH (2,2-Diphenyl-1-picrylhydrazyl), trolox (6-hydroxy-2,5,7,8-tetramethylchroman-2-carboxylic acid), and 70% EtOH, were purchased from Sigma Aldrich (St. Louis, MO, USA). Ultra-pure water was obtained from a Milli-Q water system (Millipore Bedford, MS, Boston, Massachusetts, USA).

### 3.2. Samples Analysis

For the analysis of sugars and cyclitols analysis, 0.5 g from each honey sample was measured in 50 mL plastic vials and dissolved in 25 mL of water. From each obtained solution, 5 mL were transferred to 10 mL glass vials and evaporated to dryness under a nitrogen gas flow. The obtained residuum was resolved in 2 mL of pyridine. From the obtained pyridine solution 100 μL was derivatized using TMSI (ratio 1:1) at 80 °C for 120 min. At the end of the derivatization 1 μL was taken from each sample and injected into the GC injection port. The GC-MS analysis was carried out using an AutoSystem XL gas chromatograph coupled with mass spectrometer TurboMass (both from Perkin Elmer, Norwalk, CT, USA. He at 1 mL/min was used as carrier gas. An RTX-5MS capillary column (30 m × 0.25mm × 0.250 μm, Restek, Bellefonte, PA, USA) was used. The oven temperature was programmed as follows: initial temperature of 90 °C was kept for 1 min, increased at a rate of 10.0 °C/min to 300 °C and maintained for 5 min. The injector temperature was 260 °C and injections were made in the split mode, with a split flow of 1:25. The mass spectrometer was operating as follows: ion source temperature 280 °C, ionization energy 70 eV (electron impact ionization), and *m/z* scanning range 35–650 Da. The acquisition of chromatographic data was performed by means of TurboMass (Perkin Elmer) and mass spectrum library NIST 2005 (National Institute of Standards and Technology Gaithersburg, Montgomery County, Maryland, USA).

### 3.3. Validation Parameters

Standard solutions with known concentrations were prepared and analyzed to determine the areas corresponding to each concentration. To generate calibration curves, a minimum of five concentrations of each individual standard were measured. In the cases where the determined area of peaks detected in samples did not fit the initial considered scale (it was the case of glucose and fructose), more points were added to the calibration curves. Calibration data, including retention time (Rt), retention index, calibration equations, linearity presented as a correlation coefficient (R2) of the calibration curves, limits of detection (LOD), limits of quantification (LOQ) and precision (RSD) are presented in Table 2. Retention indexes were calculated using Kovats retention index equation and established based on mixed alkane standards from C9 to C27. LOD ranged from 1.5 to 19.92 ng*mL^−1^ and LOQ from 4.55 to 60.35 ng*mL^−1^. The accuracy was evaluated as a recovery at each concentration over 80–120% of the analyte range concentrations. The results showed that average recovery at different concentration levels ranged from 93.4% to 97.2%, while the RSD was 3%. The calibration curve parameters had a good linearity, with a correlation coefficient R2 ranging between 0.9977 and 0.9988. The amount of each identified target calculated using calibration curves is highlighted in Suplementary Table S1. For the construction of calibration curves, three repetitions were realised for each concentration, and the same protocol was followed for each honey sample.

### 3.4. Determination of Physico-Chemical Properties

#### 3.4.1. Pfund Value and Honey Color

For the determination of Pfund value 4 g of honey was dissolved 8 mL of distilled water, heated up to 50 °C, and kept under stirring until the total solvation of sugar crystals was achieved. The absorbance of the obtained solution was measured with a UV-Vis spectrophotometer (NanoDrop 2000c; Thermo Fisher Scientific, Waltham, MA USA) at wave length λ = 635 nm. The honey color was determined based on Pfund scale. The Pfund value was calculated with the formula (1).

Pfund [mm] = −38.7 + 371.39 × Abs(1)

where Pfund = honey color value in the Pfund scale and Abs = absorbance at the wave length of 635 nm. 

#### 3.4.2. Acidity and pH

From each honey sample 5 g was dissolved in 37.5 mL of water. The pH of obtained solutions was measured in triplicate using a pH-meter CPC-501 (Elmetron, Chorzow, Poland) with a glass electrode. The acidity (expressed in miliequivalent of acid per kilogram of honey) was determined by the potentiometric titration of honey solution, previously prepared for pH measurement, with NaOH (0.1M) until the pH value of 8.3 was obtained. The acidity was calculated using the formula (2).

A [meq/kg] = VNaOH × 10(2)

where A = total acidity and VNaOH = the volume of NaOH (0.1M) solution used for the titration. 

#### 3.4.3. Electrical Conductivity

The electrical conductivity measurement of the honey was performed as follow: 2 g of sample was dissolved in 10 mL of deionized water. From the obtained solution 1 mL was taken, the temperature was adjusted at 20 °C, and the solution was subsequently transferred in a conductivity cell. The electrical conductivity of each sample was measured with the equipment CPC-501 equipment (Elmetron, Chorzow, Poland) in triplicate and expressed in milisiemens/centimeter.

#### 3.4.4. Antioxidant Activity

The antioxidant activity of honey was determined using the DPPH (2,2-Diphenyl-1-picrylhydrazyl) free radical scavenging assay. In brief, 5 mL of honey solution (obtained by dilution 1:50) was evaporated to dryness and redissolved in 2 mL of 70% ethanol. From ethanoic solution, 50 µL were mixed with 200 µL of 0.1 mM DPPH solution. The obtained mixtures were incubated under stirring for 30 min at room temperature in darkness. The absorbance was measured at 517 nm using a Varioskan Lux spectrophotometer (Thermo Scientific, Vantaa, Finland). The results were expressed in trolox units (6-hydroxy-2,5,7,8-tetramethylchroman-2-carboxylic acid). The equivalent antioxidant capacity was calculated using calibration curve of trolox solutions prepared at desired concentrations (between 0.025 to 0.3 μol/mL) in 70% EtOH.

### 3.5. Statistical Approach

IBM SPSS Statistical package, version 21 was used for hierarchical clustering analysis and correlation analysis. Microsoft Excel 2016 and Microsoft Power Point 2010 were used to prepare and integrate the figures composed from multiple parts.

## 4. Conclusions

The obtained results concluded that there is multiple-scale correlation between the physicochemical properties and carbohydrate contents of 18 types of honey. Consequently, it was highlighted by non-parametric tests that between different preset variables, i.e physical properties, the contents of major constituents (glucose, fructose, etc.) and minor constituents (cyclitols), demonstrated a relevant correlation. Moreover, parametric tests revealed a generally strong positive correlation between honey types, when the contents of sugars and cyclitols were assigned as variables. Nevertheless, we found that samples cultivated in 2015 and 2016 presented a distinctive correlation compared with those harvested in 2017 and 2018, due to the natural changes which occur in honey during a prolonged storage period. Hierarchical cluster analyses based on the amount of detected targets congregated the samples broadly based on the strength of the total amount. However, the dendrograms built with respect to the 23 variables selected, led to the formation of five main clusters with high significance, and six more with lower levels of significance.

## Figures and Tables

**Table 1:** Physical-chemical properties and origin of honey samples.

No	Honey Type	Pfund Value (mm)	Color According to Pfund Scale	pH	Acidity (meq/kg)	Electrical Conductivity (mS/cm)	Antioxidant Activity	Manufacturer/ Place & Origin Country/ Year
1	Bush	129.66	dark amber	4.20 ± 0.03	26.0	0.677 ± 0.03	4.43 ± 0.4	“Peter & Trisha Norris”, Lenah Valley, Tasmania, Australia, 2016
2	Leatherwood	88.81	amber	4.32 ± 0.03	21.0	0.704 ± 0.02	4.25 ± 0.05	
3	Clover	20.10	yellow- white	3.60 ± 0.01	22.0	0.255 ± 0.01	3.95 ± 0.27	
4	Multiflower	77.05	light amber	4.30 ± 0.02	12.0	0.067 ± 0.00	2.95 ± 0.16	“Fragrant Greece”, Greece, 2017
5	Multiflower	183.73	dark amber	4.15 ± 0.05	114.0	0.322 ± 0.04	4.9 ± 0.06	“Adam Gardynik”, Nyaoundere Kameron, 2018
6	Acacia	33.10	white	3.80 ± 0.02	20.0	0.103 ± 0.04	2.53 ± 0.21	Private beekeeper, Cluj County, Romania, 2017
7	Rapeseed	94.13	amber	3.66 ± 0.03	36.6	0.266 ± 0.05	4.75 ± 0.41	Private beekeeper, Cluj County, Romania, 2017
8	Multiflower	100.45	amber	3.49 ± 0.01	24.0	0.073 ± 0.01	2.74 ± 0.11	“Lume de miel”, certified by Famille Michand, France, 2018
9	Raspberry	101.56	amber	3.53 ± 0.02	30.0	0.076 ± 0.03	3.15 ± 0.19	“Medokomerc”, Čestín, Czech Republic, 2017
10	Clover	18.99	yellow- white	3.74 ± 0.04	20.0	0.046 ± 0.06	4.09 ± 0.24	“Pykoht”, Dubna, Russia, 2017,
11	Multiflower	170.76	dark amber	4.04 ± 0.02	44.0	0.140 ± 0.01	4.63 ± 0.33	“Isis Mel”, Embu-Guaçu, Brazil, 2018
12	Sunflower	62.07	light amber	3.88 ± 0.01	21.0	0.404 ± 0.04	3.8 ± 0.33	Private beekeeper, Olekszyn, Wielkopolskie, Poland, 2016
13	Raspberry	91.29	amber	3.65 ± 0.02	21.0	0.292 ± 0.03	3.48 ± 0.09	Private beekeeper, Olekszyn, Wielkopolskie, Poland, 2016
14	Buckwheat	190.7	dark amber	3.59 ± 0.03	72.5	0.556 ± 0.02	6.01 ± 0.03	“Pasieka Andrzej Kuś”, Kujawsko-Pomorskie, Poland, 2016
15	Rapeseed	34.34	white	4.02 ± 0.06	12.8	0.187 ± 0.01	3.85 ± 0.09	Private beekeeper, Solec Kujawski, Kujawsko- Pomorskie, Poland, 2016
16	Buckwheat	107.5	amber	3.89 ± 0.02	35.0	0.310 ± 0.03	5.38 ± 0.29	“Sądecki Bartnik”, Stróże, Małopolskie, Poland, 2016
17	Goldenrod	104.53	amber	3.67 ± 0.01	49.0	0.669 ± 0.05	5.36 ± 0.08	“Jakubiec gospodarstwo”, Bielsko-Biała,Śląskie, Poalnd, 2015
18	Sunflower	114.44	dark amber	3.68 ± 0.03	25.0	0.374 ± 0.04	3.91 ± 0.22	“Sądecki Bartnik”, Stróże, Małopolskie, Poland, 2016
19	Acacia	10.2	extra white	3.58 ± 0.05	22.0	0.235 ± 0.01	3.55 ± 0.2	Private beekeeper, Karczowiska Górne, Warmińsko-Mazurskie, Poland, 2017
20	Acacia	21.47	white	3.61 ± 0.02	16.5	0.213 ± 0.05	4.08 ± 0.16	Private beekeeper, Janowiec, Lubelskie, Poland 2017
21	Linden	68.01	light amber	4.02 ± 0.01	23.0	0.678 ± 0.03	4.46 ± 0.1	“Sądecki Bartnik”, Stróże, Małopolskie, Poland, 2018
22	Multifloral/linden	59.72	light amber	4.27 ± 0.03	30.0	0.854 ± 0.02	4.75 ± 0.21	Private beekeeper, Wilga Mazowieckie, Poland, 2016
23	Goldenrod	58.85	light amber	3.31 ± 0.02	42.7	0.331 ± 0.01	4.43 ± 0.06	“Słoneczna Pasieka”, Stryków, Łódzkie, Poland, 2016
24	Dandelion	101.31	amber	3.99 ± 0.00	22.0	0.050 ± 0.04	3.57 ± 0.14	Private beekeeper, Białowieża, Podlaskie, Poland, 2017
25	Ivy vine	514.55	green	3.80 ± 0.02	53.0	0.258 ± 0.01	5.29 ± 0.23	“Piotr Nowakowski”, Wrocław, Dolnośląskie, Poland, 2018
26	Buckwheat	117.04	dark amber	3.2 ± 0.01	100.0	0.100 ± 0.02	5.33 ± 0.14	Private beekeeper, Białystok, Podlaskie, Poland, 2018
27	Raspberry	87.45	amber	3.82 ± 0.03	33.0	0.061 ± 0.02	6.01 ± 0.22	Private beekeeper, Białowieża, Podlaskie, Poland 2017
28	Buckwheat	182.15	dark amber	3.78 ± 0.04	65.0	0.088 ± 0.01	6.24 ± 0.06	Private beekeeper, Białowieża, Podlaskie, Poland 2017
29	Multiflower	219.42	dark amber	3.66 ± 0.01	80,0	0.087 ± 0.05	5.62 ± 0.02	Private beekeeper, Lubelskie, Poland, 2017
30	Spring flowers	39.42	very light amber	3.60 ± 0.03	23.0	0.041 ± 0.02	2.76 ± 0.11	Private beekeeper, Janowiec, Lubelskie, Poland, 2018
31	Buckwheat	184.63	dark amber	3.59 ± 0.02	44.0	0.393 ± 0.02	5.73 ± 0.21	“Barć Świętokrzyska” Daleszyce, Świętokrzyskie, Poland, 2016
32	Honeydew	151.08	dark amber	4.49 ± 0.01	31.0	1.221 ± 0.05	5.21 ± 0.27	“Sądecki Bartnik”, Stróże, Małopolskie, Poland, 2017
33	Rape	114.07	amber	3.64 ± 0.04	15.0	0.181 ± 0.02	2.72 ± 0.19	“Sądecki Bartnik”, Stróże, Małopolskie, Poland, 2018
34	Honeydew/buckwheat	408.08	dark amber	3.58 ± 0.02	84.0	0.114 ± 0.03	7.03 ± 0.17	Private beekeeper, Karczowiska Górne, Warmińsko-Mazurskie, Poland, 2017
35	Buckwheat	160.12	dark amber	3.28 ± 0.03	95.0	0.106 ± 0.01	5.17 ± 0.07	Private beekeeper, Sosnówka, Dolnośląskie, Poland, 2018
36	Rape	85.47	light amber	3.59 ± 0.02	24.0	0.035 ± 0.02	3.48 ± 0.27	Private beekeeper, Miłków, Dolnośląskie, Poland, 2018
37	Buckwheat	231.05	dark amber	4.04 ± 0.01	29.0	0.462 ± 0.03	4.97 ± 0.28	Private beekeeper, Krzeczyn Mały k. Lubina, Dolnośląskie, Poland, 2016
38	Linden/Multiflora	76.8	light amber	4.27 ± 0.02	33.3	0.812 ± 0.03	4.21 ± 0.16	Private beekeeper, Bobrowniki, Kujawsko- Pomorskie, Poland, 2016

**Table 2:** Calibration data of detected components including: retention time (Rt), calibration equations, linearity coefficient (R2), LOD, LOQ, and precision (RSD).

Standard	Retention Time (R_t_)	Retention Index (R_i_)	Regression Equation	R^2^	RSD%	LOD (ng*mL^−1^)	LOQ (ng*mL^−1^)
Xylose	9.51	1728	y = 0.1136x − 0.0836	0.9990	2.42	10.81	32.75
	9.68	1735	y = 0.2014x + 0.0347	0.9998	2.34	19.92	60.35
D-fructose	10.08	1832	y = 0.274x − 1.3427	0.9992	1.03	8.66	26.25
	10.14	1840	y = 0.1245x − 0.5283	0.9995	2.15	3.68	11.14
	10.24	1847	y = 0.0788x − 0.5207	0.9990	1.90	3.87	11.73
D-Pinitol	10.44	1861	y = 0.4234x + 0.0917	0.9998	0.15	2.72	8.25
Quabrachitol	10.86	1878	y = 0.1074x − 0.0521	0.9993	1.08	4.12	12.47
*Allo*-inositol	10.98	1902	y = 0.4181x − 0.3954	0.9983	1.07	19.06	57.76
D-Glucose	11.15	1922	y = 0.2911x − 4.8439	0.9977	1.11	2.69	8.17
	12.25	2006	y = 0.0807x − 0.4037	0.9983	3.43	4.12	12.47
*Neo*-inositol	11.25	1934	y = 0.2969x − 0.1251	0.9995	0.93	10.89	33.00
*Muco*-inositol	11.49	1941	y = 0.2338x − 0.1819	0.9994	1.12	7.29	22.09
D-*Chiro*-inositol	11.93	1965	y = 1.279x + 0.8057	0.9994	0.10	6.02	18.25
Sequoyitol	12.01	1973	y = 0.167x − 0.0591	0.9993	1.46	7.13	21.60
Ononitol	12.12	1988	y = 0.0929x − 0.0288	0.9996	1.15	4.12	12.47
Bornesitol	12.51	2035	y = 0.2315x − 0.0338	0.9996	1.16	11.22	33.99
*Epi*-inositol	12.70	2070	y = 0.2038x + 0.1189	0.9993	0.53	5.61	17.00
*Cis*-inositol	13.00	2095	y = 0.1617x + 0.0601	0.9979	0.84	9.72	29.44
*Myo*-inositol	13.53	2120	y = 1.0889x − 0.1878	0.9997	0.56	19.42	58.88
Sucrose	18.67	2686	y = 0.0259x + 0.0075	0.9988	2.52	2.69	8.17
Maltose	18.77	2702	y = 0.1744x + 0.048	0.9981	1.86	6.16	18.65
Lactose	18.91	2730	y = 0.0151x + 0.0138	0.9987	1.90	1.50	4.55
D-(+)-turanose	19.14	2747	y = 0.1222x − 0.0637	0.9996	3.08	1.75	5.31

## Supplementary Materials

The following are available online at https://www.mdpi.com/1420-3049/25/1/34/s1, Table S1: The amount of sugars and cyclitols (in mg/mL) quantified in honey samples, where nd–not detected.
