# Supplementary material for: Correlation Study of Honey Regarding their Physicochemical Properties and Sugars and Cyclitols Content
Source: Molecules. 2019 Dec 20;25(1):34. doi: 10.3390/molecules25010034 (PMC6983052; doi:10.3390/molecules25010034)
Supplement: Supplementary file 1 [file molecules-25-00034-s001.pdf]

Table S1. The amount of sugars and cyclitols (in mg/mL) quantified in honey samples, where nd – not detected

Sample No.	Xylose	Maltose	Fructose	D-pinitol	Quebrachitol	*Allo*-inositol	Glucose	*Neo*-inositol	*Muco*-inositol	*Chiro*-inositol	Ssequitol	Ononitol	Bornesitol	*Epi*-inositol	*Cis*-inositol
1	2.52 ± 0.11	13.32 ± 0.41	368.9 ± 2.63	0.11 ± 0.01	0.15 ± 0.01	1.31 ± 0.02	308.2 ± 0.94	6.00 ± 0.04	3.57 ± 0.12	0.34 ± 0.01	0.97 ± 0.05	2.23 ± 0.11	nd	0.02 ± 0.001	nd
2	1.00 ± 0.02	17.72 ± 0.59	361.9 ± 0.45	0.04 ± 0.002	0.39 ± 0.003	1.98 ± 0.16	283.4 ± 2.84	3.99 ± 0.37	9.93 ± 0.39	0.36 ± 0.01	0.59 ± 0.03	10.6 ± 0.14	nd	0.05 ± 0.001	nd
3	1.16 ± 0.04	9.93 ± 0.13	322.6 ± 1.64	0.05 ± 0.004	9.454 ± 0.4	3.73 ± 0.05	435.1 ± 2.36	8.61 ± 0.25	4.65 ± 0.18	0.77 ± 0.04	2.60 ± 0.04	10.8 ± 0.26	0.14 ± 0.007	nd	nd
4	2.27 ± 0.07	9.52 ± 0.31	340.8 ± 0.93	0.57 ± 0.04	1.15 ± 0.07	1.14 ± 0.04	380.0 ± 2.60	3.67 ± 0.25	3.64 ± 0.25	0.50 ± 0.01	1.92 ± 0.080	17. 7 ± 0.97	nd	nd	nd
5	2.19 ± 0.06	6.87 ± 0.13	291.5 ± 1.57	0.32 ± 0.02	1.53 ± 0.18	2.56 ± 0.13	339.8 ± 0.98	7.54 ± 0.25	2.23 ± 0.07	0.51 ± 0.04	0.92 ± 0.03	25.07 ± 0.17	nd	nd	nd
6	3.69 ± 0.04	11.42 ± 0.77	343.2 ± 1.47	0.13 ± 0.003	0.12 ± 0.004	1.55 ± 0.06	290.0 ± 0.95	6.73 ± 0.13	3.68 ± 0.31	1.03 ± 0.04	2.13 ± 0.1	2.12 ± 0.32	0.05 ± 0.003	0.02 ± 0.004	0.04 ± 0.003
7	3.74 ± 0.1	17.06 ± 0.42	363± 0.93	0.37 ± 0.02	0.35 ± 0.006	1.02 ± 0.06	305.6 ± 0.95	7.36 ± 0.15	3.63 ± 0.44	0.43 ± 0.01	1.8 ± 0.05	5.83 ± 0.13	0.05 ± 0.003	0.05 ± 0.008	0.03 ± 0.009
8	1.72 ± 0.04	15.67 ± 0.34	243.3 ± 1.99	0.29 ± 0.030	4.51 ± 0.15	3.95 ± 0.19	385.4 ± 2.11	3.26 ± 0.12	1.39 ± 0.02	0.59 ± 0.02	3.01 ± 0.11	8.47 ± 0.13	nd	nd	nd
9	3.46 ± 0.04	9.87 ± 0.51	305.3 ± 1.66	0.41 ± 0.01	2.79 ± 0.05	1.64 ± 0.04	294.0 ± 0.89	6.55 ± 0.26	2.87 ± 0.06	0.41 ± 0.01	1.96 ± 0.09	6.52 ± 0.02	nd	nd	nd
10	1.37 ± 0.03	10.08 ± 0.25	402.5 ± 2.13	0.2 ± 0.005	4.37 ± 0.04	2.53 ± 0.09	315.2 ± 0.82	6.19 ± 0.09	1.42 ± 0.04	0.88 ± 0.01	4.01 ± 0.26	9.60 ± 0.20	nd	nd	nd
11	2.21 ± 0.04	16.44 ± 0.56	325.5 ± 1.62	0.56 ± 0.02	7.7 ± 0.53	4.13 ± 0.03	321.6 ± 0.59	4.06 ± 0.06	1.14 ± 0.02	0.52 ± 0.02	4.24 ± 0.2	14 ± 0.07	nd	nd	nd
12	1.55 ± 0.02	0.16 ± 0.004	376.6 ± 1.21	0.71 ± 0.002	0.29 ± 0.004	0.33 ± 0.01	285.7 ± 1.58	3.25 ± 0.29	3.26 ± 0.12	0.1 ± 0.006	0.11 ± 0.01	0.3 ± 0.006	0.02 ± 0.002	0.02 ± 0.002	nd
13	1.66 ± 0.66	5.55 ± 0.59	295.0 ± 1.75	0.13 ± 0.002	1.45 ± 0.1	0.88 ± 0.08	288.8 ± 1.08	2.48 ± 0.22	4.87 ± 0.12	0.42 ± 0.01	0.65 ± 0.04	3.29 ± 0.07	0.07 ± 0.002	0.03 ± 0.005	nd
14	2.06 ± 0.33	12.75 ± 0.39	379.9 ± 2.32	0.07 ± 0.002	0.27 ± 0.02	2.67 ± 0.18	349.3 ± 2.4	1.66 ± 0.05	4.69 ± 0.25	0.57 ± 0.02	1.01 ± 0.01	3.49 ± 0.06	0.04 ± 0.003	0.01 ± 0.002	0.04 ± 0.004
15	1.97 ± 0.06	11.84 ± 0.99	371.4 ± 0.88	0.07 ± 0.002	0.85 ± 0.03	2.24 ± 0.12	320.2 ± 1.36	8.69 ± 0.14	2.29 ± 0.06	0.82 ± 0.02	3.35 ± 0.07	2.81 ± 0.31	0.03 ± 0.002	0.02 ± 0.001	0.05 ± 0.005
16	2.13 ± 0.06	7.58 ± 0.16	409.6 ± 1.07	0.06 ± 0.001	5.79 ± 0.04	1.72 ± 0.05	350.5 ± 2.08	4.81 ± 0.17	5.15 ± 0.05	0.36 ± 0.01	1.78 ± 0.05	7.88 ± 0.08	nd	0.01 ± 0.001	nd
17	2.91 ± 0.16	15.72 ± 0.50	291.8 ± 0.59	0.07 ± 0.003	3.9 ± 0.1	1.55 ± 0.07	355.5 ± 2.11	5.88 ± 0.61	2.62 ± 0.37	0.73 ± 0.08	3.17 ± 0.04	8.35 ± 0.39	0.03 ± 0.002	nd	nd
18	3.77 ± 0.07	0.91 ± 0.02	372.1 ± 1.47	nd	1.26 ± 0.03	10.5 ± 0.18	412.8 ± 2.09	4.43 ± 0.22	7.12 ± 0.14	0.8 ± 0.06	2.43 ± 0.03	15.1 ± 0.67	nd	nd	nd

19	3.66 ± 0.09	12.36 ± 0.52	392.9 ± 2.17	0.41 ± 0.03	0.15± 0.002	0.62 ± 0.06	372.9 ± 1.18	3.82 ± 0.27	2.25 ± 0.07	0.89 ± 0.02	3.55 ± 0.06	10.2 ± 0.09	nd	nd	nd
20	3.71 ± 0.13	12.9± 0.82	366.4 ±1.55	0.55 ± 0.02	0.91 ± 0.05	2.39 ± 0.12	342.6 ± 1.34	8.44 ± 0.18	5.11 ± 0.27	0.63 ± 0.04	2.38 ± 0.03	6.5 ± 0.17	0.07 ± 0.004	nd	nd
21	1.8 ± 0.05	23.02 ± 0.15	363.2 ± 2.27	0.29 ± 0.02	0.8 ± 0.09	0.86 ± 0.02	342.9 ± 1.19	2.03 ± 0.22	1.49 ± 0.07	0.27 ± 0.01	1.24 ± 0.03	10.1 ± 0.21	nd	nd	nd
22	1.93 ± 0.07	12.54 ± 0.68	339.4 ± 1.12	0.21 ± 0.01	2.13 ± 0.16	1.16 ± 0.04	316.1 ± 1.31	3.12 ± 0.45	1.44 ± 0.08	0.18 ± 0.02	2.08 ± 0.04	5.17 ± 0.16	0.14 ± 0.002	nd	nd
23	0.85 ± 0.02	10.32 ± 0.61	311.8 ± 0.61	0.71 ± 0.04	14.92 ± 0.23	3.06 ± 0.08	379.3 ± 2.28	3.31 ± 0.35	3.08 ± 0.15	0.85 ± 0.1	nd	17.3 ± 0.29	nd	nd	nd
24	3.11 ± 0.14	9.23 ± 0.35	292.7 ± 2.29	0.41 ± 0.03	1.14 ± 0.08	1.7 ± 0.05	347.4 ± 2.63	4.12 ± 0.32	2.12 ± 0.13	0.98 ± 0.03	2.28 ± 0.05	9.21 ± 0.14	nd	nd	nd
25	3.12 ± 0.06	7.69 ± 0.4	293.6 ± 3.49	0.94 ± 0.05	6.99 ± 0.27	2.09 ± 0.12	392.1 ± 3.48	4 ± 0.03	2.48 ± 0.09	0.96 ± 0.05	4.68 ± 0.04	11.4 ± 0.03	nd	nd	nd
26	2.65 ± 0.39	12.07 ± 0.65	362.1 ± 1.68	0.84 ± 0.06	5.77 ± 0.29	2.24 ± 0.13	424 ± 3.66	5.48 ± 0.3	3.5 ± 0.08	0.95 ± 0.07	2.27 ± 0.06	4.34 ± 0.04	0.13 ± 0.001	0.03 ± 0.002	0.19 ± 0.005
27	2.7 ± 0.43	10.76 ± 0.14	286.7 ± 1.17	0.15 ± 0.006	9.35 ± 0.26	4.67 ± 0.12	383.8 ± 1.54	10.44 ± 0.75	3.31 ± 0.19	1.08 ± 0.05	3.76 ± 0.05	2.17 ± 0.13	0.06 ± 0.001	nd	nd
28	3.65 ± 0.06	14.61 ± 0.19	307.5 ± 3	0.78 ± 0.02	2.01 ± 0.14	2.4 ± 0.2	373.9 ± 1.1	4.32 ± 0.36	2.56 ± 0.03	0.35 ± 0.01	2.74 ± 0.09	11.5 ± 0.39	nd	nd	nd
29	1.37 ± 0.07	13.97 ± 0.35	303.4 ± 1.02	0.93 ± 0.03	4.93 ± 0.25	2.12 ± 0.07	449.9 ± 1.35	8.82 ± 0.13	3.77 ± 0.11	0.46 ± 0.05	1.15 ± 0.04	5.64 ± 0.13	nd	nd	nd
30	4.3 ± 0.07	5.89 ± 0.32	312 ± 1	1.49 ± 0.04	13.68 ± 0.39	11.2 ± 0.15	357.2 ± 2.31	8.6 ± 0.2	4.46 ± 0.09	0.72 ± 0.03	2.77 ± 0.02	16.6 ± 0.86	nd	0.03 ± 0.003	nd
31	3.42 ± 0.09	6.46 ± 0.06	268.4 ± 0.6	0.44 ± 0.01	0.94 ± 0.08	1.08 ± 0.11	311.7 ± 1.59	1.78 ± 0.02	2.45 ± 0.09	0.49 ± 0.01	0.82 ± 0.05	9.01 ± 0.24	0.09 ± 0.003	nd	nd
32	2.6 ± 0.08	14.94 ± 0.69	257.1 ± 1.97	1.12 ± 0.01	1.93 ± 0.1	2.12 ± 0.09	252.5 ± 1.89	6.02 ± 0.29	2.32 ± 0.08	1.07 ± 0.01	3.35 ± 0.05	22.8 ± 0.36	0.14 ± 0.004	nd	nd
33	3.88 ± 0.06	10.89 ± 0.26	299.7 ± 1.13	0.87 ± 0.04	8.07 ± 0.11	4.19 ± 0.13	391.6 ± 1.04	3.33 ± 0.18	4.36 ± 0.5	0.76 ± 0.02	1.75 ± 0.04	6.02 ± 0.23	0.14 ± 0.006	0.02 ± 0.003	nd
34	2.49 ± 0.12	9.63 ± 0.17	245.2 ± 1.31	0.13 ± 0.01	2.83 ± 0.15	2.37 ± 0.08	218.3 ± 0.77	5.88 ± 0.57	3.91 ± 0.08	0.23 ± 0.01	0.96 ± 0.04	2.2 ± 0.27	nd	nd	nd
35	2.01 ± 0.04	8.7 ± 0.16	376.6 ± 0.98	0.73 ± 0.03	2.85 ± 0.05	3.05 ± 0.01	309.8 ± 0.76	6.79 ± 0.35	3.58 ± 0.14	0.89 ± 0.03	3.66 ± 0.12	3.33 ± 0.1	nd	nd	nd
36	2.79 ± 0.03	19.7 ± 0.7	309.5 ± 1.24	0.27 ± 0.03	5.81 ± 0.01	2.03 ± 0.06	398.4 ± 4.02	1.9 ± 0.09	2.2 ± 0.08	1.38 ± 0.03	5.61 ± 0.14	17.2 ± 0.46	nd	nd	nd
37	2.60 ± 0.07	17.77 ± 0.24	349.7 ± 1.04	0.584 ± 0.02	10.17 ± 0.50	7.8 ± 0.04	360.5 ± 2.1	11.57 ± 0.7	2.96 ± 0.21	0.52 ± 0.04	3.59 ± 0.06	13.5 ± 0.23	nd	nd	nd
38	0.98 ± 0.02	8.21 ± 0.43	382.2 ± 1.66	0.15 ± 0.01	1.05 ± 0.06	0.8 ± 0.03	309.8 ± 2.05	1.83 ± 0.06	2.3 ± 0.15	0.39 ± 0.01	1.83 ± 0.03	8.58 ± 0.07	nd	nd	nd
